# The relationship of body mass index and mid-upper arm circumference with anemia in non-pregnant women aged 19–49 years in Indonesia: Analysis of 2018 Basic Health Research data

**DOI:** 10.1371/journal.pone.0264685

**Published:** 2022-03-03

**Authors:** Olwin Nainggolan, Dwi Hapsari, Christiana Rialine Titaley, Lely Indrawati, Ika Dharmayanti, Antonius Yudi Kristanto

**Affiliations:** 1 Centre for Research and Development of Public Health Efforts, National Institute Health Research and Development, Ministry of Health Republic of Indonesia, DKI Jakarta, Indonesia; 2 Faculty of Medicine, Pattimura University, Ambon, Indonesia; Universidade de Sao Paulo Faculdade de Saude Publica, BRAZIL

## Abstract

**Background and objectives:**

Anemia remains a major public health problem worldwide. This study examined the relationship between anemia and the nutritional status of non-pregnant women aged 19–49 years in Indonesia.

**Methods and study design:**

The data were derived from the 2018 Basic Health Research Data of Indonesia. We used information from 11,471 non-pregnant women aged 19–49 years. The dependent variable was anemia (hemoglobin level <12 g/dL). The independent variable was women’s nutritional status, a combined indicator of body mass index (BMI) and mid-upper arm circumference (MUAC). Our analysis controlled for women’s age, education, physical activity, fruit and vegetable consumption, and the presence of communicable or non-communicable diseases. We performed logistic regression analyses.

**Results:**

The prevalence of anemia in non-pregnant women aged 19–49 years was 22.3% (95% confidence interval [CI]: 21.4–23.3). Women with overweight and obesity were less likely to develop anemia than those with a normal BMI, regardless of their MUAC score. The highest odds for developing anemia were observed in underweight women with low MUAC scores (adjusted odds ratio [aOR] = 2.83, 95%CI: 2.19–3.68). Higher odds ratios were also observed in women with insufficient physical activity, despite their sufficient consumption of fruits or vegetables (aOR = 1.87, 95%CI: 1.06–3.28). However, women who had been diagnosed with a non-communicable disease had a reduced likelihood of developing anemia (aOR = 0.75, 95%CI: 0.67–0.83).

**Conclusions:**

Strengthening health promotion activities to improve nutritional status and healthy behaviors, particularly a healthy diet, remains important for women in Indonesia to reduce the prevalence of anemia and improve their overall health status.

## Introduction

Anemia is a condition characterized by a reduction in the number of red blood cells and/or hemoglobin (Hb) concentration, resulting in a reduced capability of the blood to carry oxygen to meet the body’s physiological needs [1]. Anemia affects half a billion women of reproductive age worldwide. In 2011, 29% (496 million) of non-pregnant women and 38% (32.4 million) of pregnant women aged 15–49 years were anemic [2]. The prevalence of anemia was highest in South Asia, as well as in the Central and West African regions [3, 4]. Several studies have identified potential risk factors for anemia, including poor nutritional status, twin pregnancy, low socioeconomic status, maternal age >30 years at the time of pregnancy, multiparity, and short birth interval [5, 6].

Anemia can occur in all age groups. However, it is commonly observed in pregnant and adolescent women [7]. Most reports indicate an increased prevalence of anemia in women, particularly due to menstrual bleeding [8]. Nevertheless, studies have reported that malnutrition and poor eating behaviors are among the most common risk factors for anemia [9–14]. Previous studies have also reported a relationship between poor nutrient intake and anemia [15].

Anemia is a major public health concern worldwide, including in Indonesia. Based on the 2018 Basic Health Research survey, the prevalence of anemia in Indonesia was 23.7% (27.2% in women and 20.3% in men) [16]. This percentage was slightly higher than that in the 2013 Basic Health Research, which reported a prevalence of anemia of 21.7% (23.9% in women and 14.4% in men) [17]. This difference might be because the iron supplementation program in Indonesia focuses mainly on pregnant women, as reflected by the low coverage of iron supplementation in other age groups, such as in adolescent girls. The 2018 Basic Health Research showed that the coverage of weekly iron supplementation in adolescent girls aged 10–19 years in the last 12 months was 76.2% (95% confidence interval [CI] 75.3–77.1). However, only 1.8% and 1.4% of adolescent girls in health facilities and school settings, respectively, consumed 52 iron tablets per year, respectively [16].

Women of childbearing age are prone to anemia due to several factors, such as blood loss during menstruation, increased physiological needs, malabsorption, insufficient iron reserves, lack of nutritional intake, hemoglobinopathy, medications, and other factors such as lifestyle and health behaviors [18]. Anemia in women of reproductive age increases the risks of complications during pregnancy or childbirth, maternal death, preterm birth, low birth weight, prenatal mortality, and antepartum or postpartum bleeding [19].

Body mass index (BMI) is an important indicator of human metabolism and nutritional status [20]. Few studies have evaluated the association between anthropometric indicators in women and anemia [21–23] and the findings of these studies are often inconsistent. Some limitations should also be noted, including the inclusion of only specific populations, such as pregnant women, obese populations, or pregnant adolescents [21, 24].

The mid-upper arm circumference (MUAC) is another common indicator used to assess nutritional status in epidemiological and clinical studies [25]. The MUAC is correlated with BMI [26]. Its measurement also requires simple equipment and does not require rigorous training. Most importantly, MUAC was reported to be an effective indicator of nutritional status when used with care and precision [27].

As most studies have reported the association between BMI and anemia among pregnant women and few have focused on non-pregnant women of reproductive age, this study examined the relationship between the nutritional status of non-pregnant women aged 19–49 years, as reflected by their BMI and MUAC, and their anemia status based on an analysis of data from the nationally representative 2018 Basic Health Research survey in Indonesia.

## Materials and methods

### Source of data and samples

The data used in this analysis were derived from 2018 Basic Health Research data. Basic Health Research is a periodic community-based survey in Indonesia implemented since 2007 to collect basic data and health indicators regarding community health conditions at national, provincial, and district levels in Indonesia. Using a standardized questionnaire, the survey collects information on the community’s health status, along with its contributing factors, based on the model described by Henrik Blum [16].

The 2018 Basic Health Research consisted of 30,000 census blocks (CBs) distributed across all 34 provinces in Indonesia. Some of the health indicators collected in this survey represented the conditions at the district level. This survey employed a two-stage sampling design. In the first stage, CBs in urban and rural areas were used as the primary sample units. In the second stage, ten households from the target population of each CB were selected with equal probability using the systematic random sampling method. Household sampling was used for the interviews and blood sample collection. Of the 30,000 CBs, 2500 CBs were selected from 106 districts for blood tests to represent blood-related indicators at the national level [16]. The present study used information from 11,471 women respondents aged 19–49 years, who were not pregnant at the time of the interview and who resided in the households selected for the blood tests.

The validation of the questionnaire used in the 2018 Basic Health Research was carried out by the *Indonesia Health Researchers Association*. The pretesting of the questionnaire was conducted by researchers from the National Institute of Health Research and Development (NIHRD), Ministry of Health, prior to data collection.

The dependent variable was anemia in non-pregnant women of reproductive age (19–49 years). Anemia was defined as a hemoglobin level of <12 g/dL [28]. The survey used Hemocue Hb 201+ devices to measure the hemoglobin levels in the blood. The system consists of a battery-operated photometer and a disposable microcuvette coated with a dry reagent that serves as a blood collection device. For the test, a drop of capillary blood was taken from the tip of the respondent’s finger or heel into the microquin. Blood in the microcuvette was analyzed using the photometer [29].

The independent variables were BMI and MUAC. Bodyweight was measured using two types of digital scales. The first type has a capacity of 50 grams to 150,000 kilograms with an accuracy of 50 grams. The second type has a capacity of 5–150 kilograms with an accuracy of 100 grams. The weighing device was calibrated daily before data collection using four 1,5 liters of bottled water. The weighing scale was placed on a hard and flat floor. Respondents were required to remove any footwear, jackets, and the contents of any pockets before standing upright on the scales.

Body height was measured using a multifunctional height meter with a capacity of 2 meters and an accuracy of 0.1 cm. After setting up the height meter by mounting it on a flat and hard wall properly, the respondents stood with the feet flat on the floor, the heels against the corner where the wall and floor meet, with the head, shoulders, and buttocks touching the wall. Respondents were asked to stand up straight with eyes looking straight ahead, with the line of sight and chin parallel to the floor. Respondents must remove any footwear, headgear, hair ponytail, or bun. When the measurer was shorter than the respondents, the measurer could stand on a bench to ensure the measurement was conducted properly. BMI was divided into three categories (underweight: <18.5 kg/m2, normal weight: 18.5–24.9 kg/m2), and overweight and obese: >25 kg/m2) [30].

MUAC was measured using a measuring tape with an accuracy of 0.1 cm. The respondent should not hold anything with relaxed arm muscles with the left sleeve rolled up to reveal the shoulder base. The measurer then determined the midpoint between the respondent’s shoulder base and the elbow. The MUAC tape was wrapped around the arm at the midpoint mark, with the arm hanging straight down. MUAC was divided into two categories according to the UNICEF 2018 guidelines (low: <23.5 cm and normal: 23.5 cm) [16].

To maintain data quality, all enumerators held at least a diploma degree in a health-related field. Multiple training sessions were also carried out starting from the training of the masters of training, followed by the training of the trainers in Jakarta and eventually the training of the data enumerators in 34 provinces throughout Indonesia. All tools used in this survey were calibrated before taking measurements. Two enumerators carried out the measurement: the measurer and the recorder. All data were entered directly on-site using CsPro version 6.1. When the data management team identified outliers, enumerators were required to repeat the measurement. Furthermore, each district was supervised by a supervisor and an external validator team.

### Other potential predictors

Our analyses included several potential predictors (women’s age, education level, physical activity, consumption of fruits and vegetables, and the presence of communicable or non-communicable diseases) that were considered confounding variables. The respondents were divided into three age categories (19–24, 25–35, and 36–49 years). The education variable was divided into three categories (primary school, junior high school, and university).

The respondents’ physical activity was measured out according to the modified Global Physical Activity Questionnaire (GPAC), which is part of the WHO STEPS instrument for measuring and monitoring risk factors for non-communicable diseases [31]. The metabolic equivalent of each task, which was the ratio of a person’s working metabolic rate relative to their resting metabolic rate, was used to determine if the respondent had sufficient physical activity per week [32]. Insufficient and sufficient physical activity was defined as metabolic equivalent of the task of <600 and ≥600 per week, respectively [33].

Fruit and vegetable consumption were measured based on the frequencies and serving sizes of fruits and vegetables ingested by household members aged ≥5 years. The instrument used to collect vegetable and fruit consumption data was based on the WHO STEPS [34]. In this analysis, we calculated the number of days that fruits and vegetables were consumed per week and the average number of servings per day. Sufficient and insufficient consumption were defined as the consumption of at least five or less than five servings of vegetables and fruits per day for seven days a week, respectively.

Regarding non-communicable diseases, the respondents were asked whether they had ever been diagnosed with asthma, cancer, diabetes mellitus, heart disease, hypertension, stroke, chronic renal failure, or joint disease by health professionals. Respondents who answered "yes" to any of these diseases were categorized as having a non-communicable disease. Similarly, respondents reporting having ever been diagnosed by health professionals with one of the communicable diseases were categorized as having a communicable disease. These communicable diseases were acute respiratory infections (in the last month), pneumonia (in the last year), pulmonary tuberculosis, hepatitis (in the last year), diarrhea (in the last month), malaria (in the last year), and filariasis (in the last 3 years).

### Data analysis

In the first stage, a descriptive analysis was performed to examine the frequency distributions of all variables used in the analysis. Contingency tables were also used to examine the distributions of variables based on the women’s nutritional status. This analysis was followed by the bivariable and multivariable logistic regression analyses to assess the relationship between the combination of BMI and MUAC with anemia status, before and after adjusting for potential confounders. The multivariable logistic regression analysis included all variables that were significantly related to anemia status in the bivariable analysis.

Analyses were performed by considering the complex survey design, including the averaged weight variables, primary sampling unit, and strata. The amount of sample generated using complex sample analysis was defined as the number of weighted N. The final model used in this analysis was the model including all variables significantly associated with anemia status, using a significance level of 0.05 as the cut-off point. The odds ratios (ORs) and 95% confidence intervals (CIs) of all variables in the final model were reported. All analyses were performed using SPSS version 15 (SN: 6a45501054a0b0c57002).

### Ethics approval

Ethics approval for the 2018 Basic Health Research of Indonesia was obtained from the Health Research Ethics Committee of the National Institute of Research and Development, Ministry of Health of the Republic of Indonesia (No: LB.02.01/2/KE.267/2017 and LB.02.01/2/KE.024/2018). All respondents were asked to sign an informed consent form before the interview was conducted and blood samples were collected.

## Results

Our study used information collected from 11,471 non-pregnant women aged 19–49 years from all provinces of Indonesia. Overall, 22.3% of the non-pregnant women of reproductive age showed anemia (95% CI: 21.4–23.3%). The prevalence of chronic energy deficiency based on a BMI of <18.5 kg/m^2^ (underweight) was 4.3% (95% CI: 4.2–4.4%) and a MUAC of <23.5 cm was 7.8% (95% CI: 7.7–7.0%).

Table 1 shows the basic sociodemographic characteristics of the respondents included in this study. More than 54% of respondents lived in urban areas, most were married, and more than 50% had graduated from junior high school or above. The highest proportion of respondents were aged 36–49 years. Based on the women’s status of anemia, we identified a significant association of age and education level with anemia. Therefore, the multivariable model included women’s age group and education level.

**Table 1:** Socio-demographic characteristics of the study respondents. The 2018 Basic Health Research of Indonesia.

Variable	N	%	95% CI		Anemia Status (%)		
					No	Yes	P-value
**Type of residence**							0.94
Urban	6250	54.5	53.6	55.4	77.6	22,4	
Rural	5221	45.5	44.6	46.4	77.7	22,3	
**Age (**year’s old)							0,00
19–24	862	7,6	7,1	8,1	57.8	42.2	
25–35	3917	34,4	33,4	35,3	80.8	19.2	
36–49	6614	58,1	57,1	59,0	78.8	21.2	
**Marital status**							0.41
Married	10773	93.9	93.4	94.4	77.8	22.2	
Separated/divorced	360	3.1	2.8	3.5	75.1	24.9	
Widowed	338	2.9	2.6	3.3	75.8	24.2	
**Education level**							0,00
Low	5367	46.8	45.6	48.0	79.6	20.4	
Middle	5462	47.6	46.5	48.8	76.3	23.7	
Higher	642	5.6	5.1	6.1	73.0	27.0	
**Occupation**							
Housewife	6393	55.7	54.5	56.9	77.0	23.0	0,37
Still enrolled in school	71	0.6	0.5	0.8	78.3	21.7	
Civil employee/army/state-owned enterprise employee	93	0.8	0.7	1.0	76.7	23.3	
Private employee	628	5.5	5.0	6.0	75.2	24.8	
Entrepreneur	1463	12.8	12.1	13.5	77.9	22.1	
Farmer	1304	11.4	10.6	12.2	80.1	19.9	
Fishery worker	8	0.1	0.0	0.1	80.2	19.8	
Informal worker	759	6.6	6.0	7.3	78.7	21.3	
Other	750	6.5	5.9	7.2	79.8	20.2	

The distributions of respondents according to the different characteristics examined in the study are shown in Table 2. According to women’s nutritional status, the highest proportion of respondents were overweight/obese with normal MUAC (53.2%, 95%CI: 52.1–54.2%), followed by women with normal MUAC and BMI (38.4%, 95%CI: 37.4–39.4%). Approximately half of the respondents had never been diagnosed by health workers with communicable or non-communicable diseases, whereas around 38% reported non-communicable diseases only. Most respondents (78.4%, 95%CI: 77.2%-79.5%) had sufficient physical activity yet insufficient consumption of fruits and vegetables.

**Table 2:** Respondent characteristics based on the variables analyzed in this study. The 2018 Basic Health Research of Indonesia.

Variable	N	%	95% CI		Anemia Status (%)		P Value
					No	Yes	
**Anemia**							
No	8910	77.70	76.70	78.60			
Yes	2561	22.30	21.40	23.30			
**Women’s BMI/MUAC**							0,000
Overweight/obesity + normal MUAC	5974	53.20	52.10	54.20	81.7	18.3	
Normal BMI + normal MUAC	4313	38.40	37.40	39.40	75.4	24.6	
Underweight + normal MUAC	117	1.00	0.90	1.30	74.3	25.7	
Overweight/obesity + low MUAC	17	0.20	0.10	0.30	79.3	20.7	
Normal BMI + low MUAC	497	4.40	4.00	4.90	68.8	31.2	
Underweight + low MUAC	318	2.80	2.50	3.20	56.2	43.8	
**Presence of communicable and non-communicable disease**							0,000
Healthy	5604	48.90	47.80	49.90	74.9	25.1	
Communicable disease only	812	7.10	6.60	7.60	72.7	27.3	
Non-communicable disease only	4320	37.70	36.60	38.70	81.8	18.2	
Both communicable and non-communicable diseases	735	6.40	5.90	7.00	79.7	20.3	
**Healthy behaviour**							0,014
Sufficient physical activity + sufficient fruits/vegetable consumption	387	3.40	3.00	3.80	78.8	21.2	
Sufficient physical activity + insufficient fruits/vegetable consumption	8994	78.40	77.20	79.50	78.2	21.8	
Insufficient physical activity + sufficient fruits/vegetable consumption	85	0.70	0.60	0.90	65.4	34.6	
Insufficient physical activity + insufficient fruits/vegetable consumption	2004	17.50	16.40	18.60	75.7	24.3	

The frequency distributions of all variables examined in this study according to women’s anemia status are also presented in Table 2. The highest percentage of anemia was observed in women who were underweight and had a low MUAC (43.8%). Interestingly, we found that the percentage of anemia was the lowest in women who were overweight/obese with normal MUAC (18.2%), followed by overweight/obese women with low MUAC (20.7%). According to the history of illnesses, the lowest percentage of anemia was observed in women who had been diagnosed with a non-communicable disease only (18.2%) compared to the other categories. The importance of physical activity and consumption of fruits and vegetables was also apparent. Women with sufficient physical activity and fruit and vegetable consumption had the lowest anemia percentage compared to that in women in the other categories.

The results of the multivariable analyses are shown in Table 3. We observed a significant association between women’s anemia status and their BMI, MUAC (*p*<0.001), and disease history (*p*<0.001). In general, women with overweight and obesity were less likely to develop anemia than women with normal BMI, regardless of their MUAC. Compared to women who were obese and had normal MUAC, women with normal BMI and MUAC had higher odds of developing anemia (OR = 1.37, 95%CI: 1.23–1.52). The odds were even greater in women with normal BMI and low MUAC (OR = 1.81, 95%CI: 1.45–2.26). The highest odds were observed in women who were underweight and had a low MUAC (OR = 2.83, 95%CI 2.19–3.68).

**Table 3:** Multivariable analysis of the associations between anemia and nutritional status, presence of illness, and healthy behaviors in non-pregnant women aged 19–49 Years. The 2018 Basic Health Research of Indonesia.

Variable	β	aOR	(95% CI)		*P*
**Age (year’s old)**					*0*,*000*
25–35		*Ref*			
19–24	1.04	2,82	(2,36–3,37)		
36–49	0.24	1,28	(1,14–1,44)		
**Education level**					*0*.*001*
Higher		Ref			
Low	-0.37	0.69	(0.55–0.86)		
Middle	-0.25	0.78	(0.63–0.97)		
**Women’s BMI/MUAC**					*0*.*001*
Overweight/obesity + normal MUAC		*Ref*			
Normal BMI + normal MUAC	0.31	1.37	(1.23–1.52)*		
Underweight + normal MUAC	0.35	1.42	(0.92–2.19)		
Overweight/obesity + low MUAC	0.18	1.20	(0.37–3.93)		
Normal BMI + low MUAC	0.59	1.81	(1.45–2.26)*		
Underweight + low MUAC	1.04	2.83	(2.19–3.68)*		
**Healthy**		*Ref*			*0*,*001*
Communicable disease only	0.09	1.10	(0.91–1.33)		
Non-communicable disease only	-0,29	0.75	(0.67–0.83)*		
Both communicable and non-communicable diseases	-0.12	0.89	(0.73–1.10)		
**Healthy behaviour**					*0*.*056*
Sufficient physical activity + sufficient fruits/vegetable consumption		*Ref*			
Sufficient physical activity + insufficient fruits/vegetable consumption	0.01	1.01	(0.76–1.33)		
Insufficient physical activity + sufficient fruits/vegetable consumption	0.63	1.87	(1.06–3.28)*		
Insufficient physical activity + insufficient fruits/vegetable consumption	0.10	1.10	(0.82–1.49)		

Note: *) p<0.*05*.

Furthermore, in this study, women who had been diagnosed by health workers with a non-communicable disease had significantly reduced likelihood of developing anemia compared to women without any such illnesses (OR = 0.75; 95%CI: 0.67–0.83). Women who had insufficient physical activity and sufficient fruit/vegetable consumption had a significantly higher odds of having anemia compared to women with sufficient physical activity and fruit/vegetable consumption (OR = 1.87, 95%CI: 1.06–3.28).

## Discussion

### Main findings

The results of our study showed that anemia remains a public health problem in Indonesia among non-pregnant women of reproductive age (19–49 years). We observed a significant association between anemia and women’s nutritional status, history of illness, and healthy behavior. Women with overweight and obesity were less likely to develop anemia compared to women with a normal BMI, regardless of their MUAC scores. We also found an increased likelihood of developing anemia in women with insufficient physical activity and sufficient fruit/vegetable consumption. Moreover, women diagnosed with a non-communicable disease had a reduced likelihood of developing anemia compared to women who had never been diagnosed with any illnesses. Our findings emphasize the need for effective health promotion interventions, including health education programs, to improve the nutritional status and healthy behaviors of women to reduce the prevalence of anemia and increase women’s health status in general.

### Anemia and chronic energy deficiency in women of reproductive age

The high prevalence of anemia in non-pregnant women aged 19–49 years in this study indicated that anemia remains a public health problem [35]. Consequently, anemia is an important issue that requires serious attention, particularly as its prevalence has been relatively stagnant over time. In 2011, data from the World Health Organization (WHO) showed a prevalence of anemia in Indonesia of 22% (95% CI 12–37) [28].

Apart from anemia, women of childbearing age often suffer from chronic energy deficiency [36]. The 2103 Basic Health Research showed a prevalence of chronic energy deficiency in non-pregnant women of reproductive age of 14.5%, which increased to17.3% in pregnant women [17]. Studies from other countries have also reported a high prevalence of chronic energy deficiency in women of reproductive age [37, 38]. These findings signify the need for evidence-based and effective health interventions targeting all women of reproductive age to reduce and prevent anemia. Women, including adolescent girls, should be encouraged to consume iron supplementation, particularly during the menstrual period [39]. Moreover, health promotion programs to ensure the consumption of balanced and varied diets, including counseling by health workers and community workers, will also be beneficial [40, 41].

### Factors associated with anemia in women of reproductive age

The association between anemia status and BMI and MUAC among the women included in our analysis is consistent with findings from other studies. A study conducted in 2013 reported a significant downward trend of anemia along with increased BMI; additionally, compared to women of normal weight, overweight and obese women also had lower odds of anemia [42]. Moreover, an inverse correlation between anemia and central obesity (PR = 0.75; 95% CI 0.63–0.89) was also reported. Similarly, a review on obesity and iron deficiency also reported low iron concentrations in the adult population with a high BMI [43]. Another study from Ethiopia also reported that MUAC of more than 23 cm reduced the incidence of anemia incidence by 0.41 [44].

A study conducted in Mexico, Peru, and Egypt reported similar findings [45]. The proportions of individuals with anemia in the group of women who were overweight (25 kg/m^2^ ≤BMI <30 kg/m^2^) and obese (BMI> 30 kg/m^2^) were similar among the three countries. The prevalence of anemia in women from Egypt decreased as BMI increased. Although the difference between anemia prevalence was not significant across BMI groups among women in Peru, a decreasing trend of anemia in women was also observed with increasing BMI [45]. A high-calorie diet and adequate intake of animal-based food may increase hemoglobin formation in women with obesity [46]. A previous study with school children in China reported that although children with obesity had high hemoglobin levels, they could experience iron deficiency, which was not analyzed in our study [46].

Despite research results demonstrating a positive relationship between overweight and obesity with a reduced incidence of anemia, several studies have reported obesity as a cause of anemia in women [47]. Low serum iron levels are associated with weight gain and increased BMI [48]. Additionally, low iron levels and anemia also lead to fatigue and reduced physical activity contributes to weight gain [49].

Our findings showed a lower likelihood of developing anemia among women with non-communicable diseases compared to women who did not have any diseases. Previous studies reported increased risks of non-communicable diseases, such as diabetes mellitus, in individuals with excess iron in the body [50, 51]. We also postulated that the reduced risk of anemia in women with non-communicable diseases might be due to a high awareness of nutritional status and health conditions as a result of these women’s frequent encounters with health professionals. This frequent contact could help women to prevent nutritional-related problems and allow the early detection of health problems.

Furthermore, our results showed a stronger relationship of adequate physical activity and fruit and vegetable consumption with the occurrence of anemia. This finding is consistent with those of other studies reporting high risks of developing anemia among less active women [52]. Additionally, reverse “causation” might also contribute to this condition, as women with anemia might reduce their physical activities due to anemia signs and symptoms.

One explanation for the non-significant relationship between fruit and vegetable consumption and anemia was how this variable was constructed in our analysis. Adequate fruit and vegetable consumption was determined solely from the serving sizes of fruits and vegetables. The survey did not have information about the type of fruits and vegetables, which could be used to identify the consumption of iron-rich foods. In our study, the cut-off point used to define sufficient consumption was based on the WHO recommendations to prevent non-communicable diseases [53]. Therefore, our findings indicate the need to investigate the most appropriate cut-off point to prevent other health issues, such as anemia.

In general, our findings emphasize the necessity to strengthen iron supplementation programs for all women of reproductive age. Strategies to promote physical activity should be prioritized to improve community health behaviors. Our findings also demonstrate the need for a comprehensive assessment of the nutritional status of the women in the survey to help in the early detection of the double burden of malnutrition.

### Strengths and limitations

This is the first study in Indonesia to investigate the relationship between combined BMI and MUAC and anemia in non-pregnant women of reproductive age. Since we used nationally representative data, our findings can be used to design and implement evidence-based interventions to prevent and reduce anemia at the national level. However, this study has several limitations. As in other cross-sectional surveys, the answers provided by the women were based on their recall ability. In the survey, data were collected manually and the calculation of anthropometric measurements such as BMI was conducted later after the fieldwork. Consequently, any inconsistencies between BMI and MUAC assessments could not be re-validated directly in the field at the time of data collection.

## Conclusions

Overall, our study results highlight the important role of BMI, MUAC, physical activity level, balanced diet, and history of disease in the risk of anemia among women of reproductive age. Educational interventions to improve the nutritional status of women are required. Interventions aiming to prevent anemia should target both pregnant and non-pregnant women of reproductive age. Furthermore, efforts to promote a healthy diet and lifestyle will be beneficial for improving the nutritional status of women in Indonesia.

## Supporting information

S1 File Instrument in English and Indonesia. (ZIP)

S2 File Statement of data restrictions. (PDF)
