# Supplementary material for: The relationship of body mass index and mid-upper arm circumference with anemia in non-pregnant women aged 19–49 years in Indonesia: Analysis of 2018 Basic Health Research data
Source: PLoS One. 2022 Mar 3;17(3):e0264685. doi: 10.1371/journal.pone.0264685 (PMC8893704; doi:10.1371/journal.pone.0264685)

**Instrumen Perilaku makan buah dan sayur**


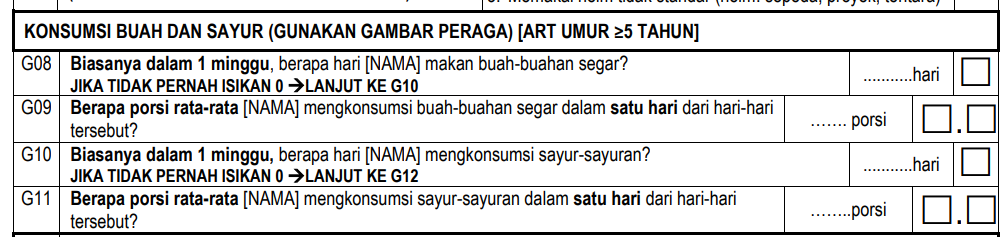


**Instrumen Pengukuran Aktivitas Fisik**
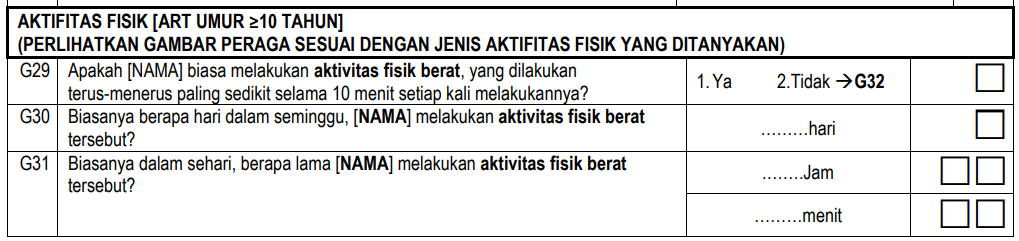


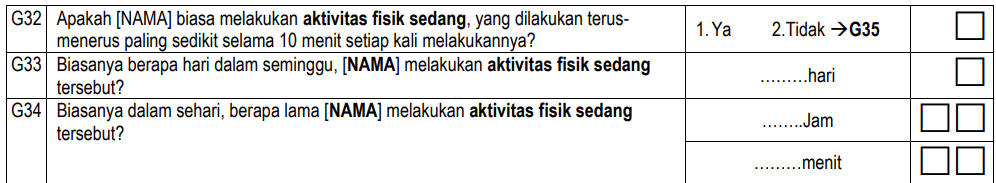


**Instrumen Pengukuran Tinggi Badan dan Berat Badan**


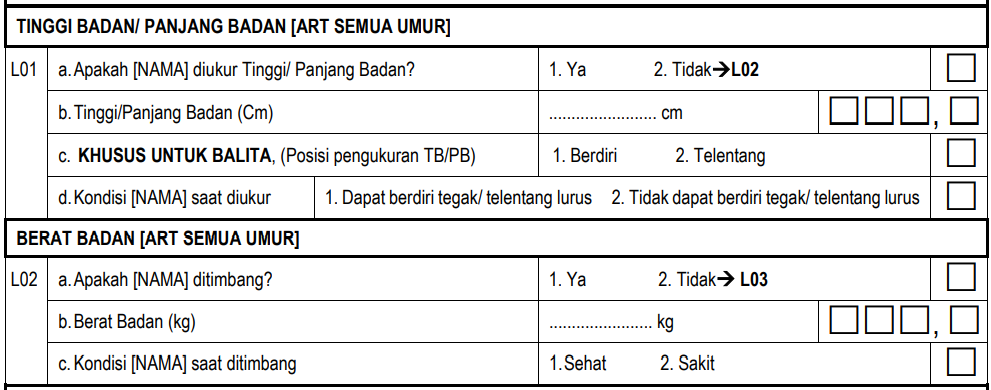


**Instrumen Pengukuran Lingkar Lengan Atas**


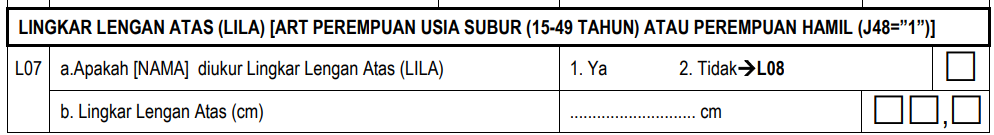

Supplement: S1 File — (ZIP) [file pone.0264685.s001.zip › Instrument in English and Indonesia/INSTRUMENT PERILAKU DAN PENGUKURAN IND.docx]
