# Supplementary material for: The relationship of body mass index and mid-upper arm circumference with anemia in non-pregnant women aged 19–49 years in Indonesia: Analysis of 2018 Basic Health Research data
Source: PLoS One. 2022 Mar 3;17(3):e0264685. doi: 10.1371/journal.pone.0264685 (PMC8893704; doi:10.1371/journal.pone.0264685)
Supplement: S2 File — (PDF) [file pone.0264685.s002.pdf]

KEMENTERIAN KESEHATAN RI

BADAN PENELITIAN DAN PENGEMBANGAN KESEHATAN

Jalan Percetakan Negara No. 29 Jakarta 10560 Kotak Pos 1226

Telepon : (021) 4261088 Faksimile : (021) 4243933

Website http://www.litbang.depkes.go.id E-mail sesban@litbang.depkes.go.id

SURAT PERNYATAAN

Nomor : IR.03.01/1/4375/2020

Pada hari ini ...Selasa... Tanggal ...20... Bulan ...10... Tahun ...2020... yang bertanda tangan di bawah ini :

Nama	: Olwin Nainggolan, S.Si., MKM.
Jenis Kelamin	: Laki-Laki
Alamat E-mail	: olwin.n@gmail.com
Hp	: 085214145673
NIP/NIM/NPM	: 196803141998031001
Pekerjaan	: PNS
Instansi	: Puslitbang Upaya Kesehatan Masyarakat
Alamat Instansi	: Jl. Percetakan Negara No.29 Jakarta Pusat 10560
Judul Penelitian	: Profil Wanita Usia Subur (WUS) terhadap Masalah Anemia di Indonesia (Analisis Data Riskesdas 2018)

Menyatakan dengan sesungguhnya bahwa :

1. Saya sanggup dan bersedia untuk mematuhi ketentuan - ketentuan yang telah ditetapkan dalam melakukan kegiatan penelitian dan pengembangan sesuai dengan Undang – Undang Kesehatan Nomor 36 Tahun 2009 tentang Kesehatan dan Peraturan Pemerintah Nomor 39 Tahun 1995 tentang penelitian dan Pengembangan Kesehatan.
2. Saya telah menerima subset data hasil penelitian Riskesdas 2018 milik Badan Penelitian dan Pengembangan Kesehatan.
3. Data hasil penelitian yang saya peroleh sebagaimana dimaksud pada poin 2 akan saya pergunakan hanya/terbatas untuk kepentingan Artikel, sehingga saya :
   a. Tidak akan membuat salinan dari data tersebut untuk keperluan lain dan pihak lain atau mengalihkan data tersebut kepada pihak lain.
   b. Akan mempergunakan data tersebut hanya untuk 1 (satu) topik judul penelitian, sesuai dengan persetujuan yang diberikan secara formal oleh Badan Litbang Kesehatan.
   c. Apabila saya menggunakan data untuk keperluan lain selain dari ketentuan di atas harus mengajukan kembali secara formal kepada Kepala Badan Litbang Kesehatan.
   d. Akan melakukan komunikasi dengan pihak Laboratorium Manajemen Data untuk pemahaman variable subset data.
   e. Untuk melakukan publikasi hasil analisis, saya sanggup dan bersedia untuk terlebih dahulu memperhatikan etika dan manfaat bagi kepentingan masyarakat.
4. Saya berkewajiban untuk menyerahkan hasil analisis kepada Laboratorium Manajemen Data Badan Penelitian dan Pengembangan Kesehatan.

Demikian surat pernyataan ini saya buat dengan sesungguhnya tanpa adanya unsur paksaan dari pihak manapun. Apabila dikemudian hari terjadi penyimpangan dari pernyataan saya tersebut, maka hak penggunaan data dan publikasi dinyatakan batal demi hukum, serta tidak dapat mengajukan kembali permohonan penggunaan data-data Badan Litbangkes untuk kepentingan apapun.

Mengetahui,	Penerima Data
Sekretaris Badan Litbangkes	METERAI TEMPEL AA075AHF664054920 6000 ENAM RIBU RUPIAH
Dr. Nana Mulyana	

*)Pembuat set data Olwin Nainggolan, S.Si., MKM.
